# The potential immunomodulatory effect of levamisole in humans and farm animals

**DOI:** 10.5455/javar.2023.j717

**Published:** 2023-12-31

**Authors:** Mohammad Hossein Gholami, Ali Rassouli, Sepideh Mirzaei, Farid Hashemi

**Affiliations:** 1 Faculty of Veterinary Medicine, Islamic Azad University, Kazerun, Iran; 2 Department of Comparative Biosciences, Faculty of Veterinary Medicine, University of Tehran, Tehran, Iran; 3 Department of Biology, Faculty of Science, Islamic Azad University, Science and Research Branch, Tehran, Iran

**Keywords:** Levamisole, anthelminthic, immunomodulatory, adjuvant

## Abstract

This study conducted a literature review to investigate the immunomodulatory effect of levamisole in both humans and farm animals. The following procedure was followed for database searching: PubMed, Google Scholar, Web of Science, and Cochrane Library. All research works were updated to September 2022. The terms used in the literature search were included: (“levamisole” OR “immunity” OR “immune system”) AND (“adjuvant” OR “fish” OR “poultry” OR “farm animal” OR “cattle” OR “sheep”). The current review enlightens the extensive potential of levamisole as an adjuvant immunotherapeutic agent and explains its divergent applications beyond its antiparasitic use as an adjuvant, dietary supplement, immunostimulant, antiviral, and anti-cancer drug in humans and farm animals. In the articles examined, various mechanisms have been proposed for levamisole immunoprotective effects, but hormonal alteration and stress hormone reduction are indicated as the main mechanisms in various animal species.

## Introduction

Intestinal parasites have a significant impact on the health of both humans and domestic animals, leading to notable economic consequences. Helminth-related diseases are estimated to affect approximately one-third of the global population, particularly in developing countries [1]. Intestinal parasite infection can induce high morbidity and low mortality in domestic animals and humans [2]. Co-infections of parasites with specific bacteria, such as *Helicobacter pylori*, have been reported to aggravate gastrointestinal and systemic diseases in humans and animals [3].

In African countries, a higher prevalence of parasitic diseases in children was reported due to the climatic conditions and the low level of health status [4]. Iron deficiency anemia, abdominal pain, and low weight gain were reported as harmful effects of parasitic infections in the body [5–7].

**Figure 1.**
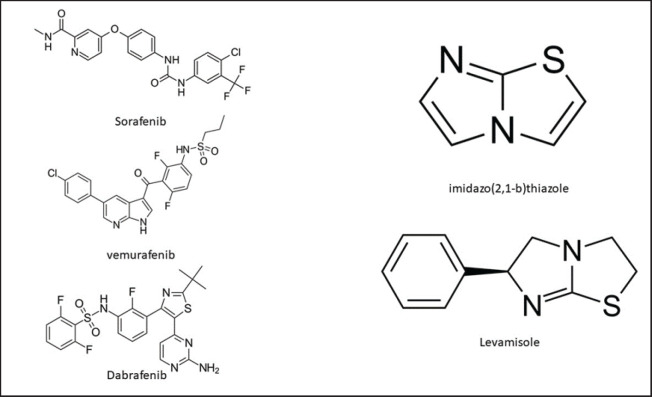
Levamisole, imidazo(2,1-b) thiazole, and some derivative compounds of an anticancer family substitute.

Levamisole, an old but useful antiparasitic, is used for the treatment and prophylaxis of a number of parasitic infections [8]. It was developed as an anthelmintic drug in 1966 and was originally used in human and animal species for the treatment of *Trichuris trichiura*, *Ascaris lumbricoides*, and hookworm infections [9]. It is an important member of the imidazothiazole derivatives with little drug resistance after one decade of continuous use (Fig. 1) [10].

In spite of the therapeutic efficacy and medical advantages of levamisole, the Food and Drug Administration banned its usage in 1998 for humans because of its severe side effects, such as rash and agranulocytosis [11]. However, it is still used in developing countries, including Iran [12]. Levamisole’s immunomodulatory effect occurs through its effect on both stimulatory and inhibitory influences on the immune system. Furthermore, the anticancer influence of levamisole was indicated by its influences on various mechanisms of cancer cells, including interferons, lymphocyte cycle, energy consumption, and granulocytopenia [13].

Levamisole has been extensively utilized for its immunomodulatory properties in various diseases, such as rheumatoid arthritis, where it is often administered in combination with 5-fluorouracil. In addition, it has shown promise in patients with colon cancer or melanoma [14]. Levamisole was used as an adjuvant for oral cancer radiotherapy, which improved immunity by increasing lymphocyte subpopulations [15]. Recently, the positive influence of levamisole on infectious viruses, especially coronavirus disease 2019 (COVID-19), has been suggested [16].

In this review, we will review the beneficial effects of levamisole as an immunomodulatory agent in various diseases and conditions. The goal is to indicate levamisole’s influence on the promotion of the innate immunity system as well as the humoral immune response in humans and farm animals.

## Materials and Methods

Various studies have been conducted on levamisole‘s influence on immunity as well as different bacterial and viral diseases. The following procedure was followed for database searching: PubMed, Google Scholar, Web of Science, and Cochrane Library. All research works were updated to September 2022. The terms used in the literature search were included: (“levamisole” OR “immunity” OR “immune system”) AND (“adjuvant” OR “fish” OR “poultry” OR “farm animal” OR “cattle” OR “sheep”). Studies were included based on their quality and rate of relevancy. Differences in inclusion and exclusion criteria were resolved through the collaboration of the authors to reach an agreement.

## Results and Discussion

### Levamisole’s impact on the immune system

A wide range of compounds have been employed as immunostimulatory agents in diverse conditions. While the primary purpose of using immunomodulators is to prevent and treat infectious diseases, they are also utilized to address stress-induced immunosuppression, promote neonatal immune system maturation, and develop strategies to minimize the metabolic burden associated with initiating an immune response [17]. Levamisole is well known to have immunomodulatory properties, but the influence of levamisole on various animals has not been sufficiently clarified [18].

Dietary usage of levamisole as an immunomodulator in farm animals and the fish production industry has been suggested as a counterbalance for antimicrobial overuse. The consumption of antibiotics has been extremely increased in recent decades, and their negative effects on human and animal health have already been reported [19–21].

In fish species, the immunostimulatory potential of levamisole is suggested to be because of improvements in leukocyte production [22], an increase in the rate of oxidative radical production [20], promotion of phagocytosis [23], induction of cytotoxic cells [24], promotion of the oxidative stress cycle in the cells [20], stimulation of lysosomal activities [25–27], and induction of the complement system [26], although its exact mechanisms of action are not well understood. In aquaculture, the dose-dependent effect of levamisole has been suggested, and it seems that the drug mainly affects the innate immune system. Levamisole was well absorbed after oral administration (100 mg/kg) for five consecutive days reducing stress indicators such as cortisol levels and promoting the immune system in belugas [28]. Pahor-Filho et al. [29] also reported that 15 days of oral administration of levamisole reduced cortisol levels amid stress responses and promoted immunity against *Aeromonas hydrophila* infection in fish (*Piaractus mesopotamicus*) [29]. It is worth noting that both studies showed similar results when assessing the respiratory burst of leukocytes, complement system hemolytic activity, activity of serum lysozyme, and plasma cortisol levels. Altogether, levamisole reduced stress and improved the innate immune system in different fish species. Most of the positive effects of levamisole seem to be the consequence of hormonal alteration in fish through the nervous system [29]. Recent research in mice and fish reported the immunostimulatory effect of levamisole could be induced by decreasing corticosterone levels [29,30]. Further studies have been considered to investigate compounds containing levamisole in fish, and adding levamisole to the fish diet has been suggested.

The humoral immune response to levamisole was also promoted in neonatal Jersey calves by weekly levamisole administration. Levamisole HCl use in the first hour after birth (3 mg/kg) in calves significantly altered serum cholesterol, LDL, HDL, triglycerides, cortisol, and white blood cells (WBCs) within the normal reference range [31]. In another study, levamisole administration by oral route (2 mg/kg) was evaluated in 30 1–2-day-old Holstein calves three times every other day. Oral administration of levamisole had no significant change in the main parameters, such as hematocrit, WBCs, differential leukocyte count, total serum protein, and disease occurrence, when compared to the control group. However, the quantity of neutrophils and monocytes was significantly different, as were the levels of gamma globulin, in comparison to the control group on days 14, 21, and 28, respectively [32].

Moreover, the immunostimulatory effect of levamisole supplementation was examined in periparturient Holstein-Friesian crossbred dairy cows at the late gestation stage. It significantly improved blood antioxidant parameters, including malondialdehyde, glutathione peroxidase, glutathione-S transferase, and superoxide dismutase, after using four doses of levamisole before parturition. Serum and colostrum total immunoglobulin (Ig) G levels after parturition improved in levamisole-treated groups [33]. However, two doses of levamisole administration in pre-parturient Jersey and Holstein dairy cows did not improve blood antioxidant parameters or postpartum reproductive performance [34]. The positive effect of levamisole on the reproductive system of cattle has been detected by an improvement in fertility rate after insemination. Not only did the rate of uterine infection decrease after artificial insemination but also the level of plasma progesterone before pregnancy dramatically increased with the stimulatory dose of levamisole [35].

In another study, the immunostimulatory potential of levamisole administration on day 1 was detected in lambs with higher weight gain in the treatment group [36]. The combination of levamisole with a number of ovine vaccines resulted in increased short- and long-term immunoprotection, which was evaluated by examining the main parameters in blood samples [37,38].

Levamisole’s impact on the immune system of weaned pigs has been thoroughly examined and extensively studied [39–42]. Oral or injectable administration of levamisole has been studied for weight gain, mucosal protection by assessment of IgA status, and humeral immune stimulation at different ages in pigs [41]. Concomitant use of levamisole with viral and bacterial vaccines in pigs has shown that levamisole not only improves antibody production but also increases the half-life of antibodies in pigs [43–46]. Interestingly, levamisole reduced cortisol levels after experimentally induced stress on pigs for 16 days. A reduction in the recovery period of the immune system, detected after levamisole administration 1 week after the termination of stress, proved that levamisole can act as an anti-stress drug [47]. Collectively, the greatest effect of levamisole in large and small ruminants can be attributed to its effect on the susceptible stages of immune system function, including the parturition period as well as neonatal immunity and well-being. It seems that the impact of levamisole on the level of immunity and the subsequent reduction of stress hormones has improved the immune system in cattle and sheep, which has been confirmed by blood tests [36,48]. More extensive studies on the effect of levamisole synergist use with other substances in large and small ruminants are suggested. In addition, a comparison of the continuous usage of levamisole in the short-term safety-sensitive period of the diet compared to injection is also suggested. Recent studies have investigated the manufacturing of nanomedicines based on highly toxic compounds [49]. Due to the high sensitivity of cattle and sheep, the production of nanolevamisole and its investigation in cattle and sheep are also recommended.

The beneficial effects of levamisole on poultry immunity, disease control, and vaccination benefits in industrial poultry production have been widely suggested. Many diseases in poultry debilitate the immune system, leading to the use of high amounts of antibacterial drugs to reduce the morbidity and mortality rates of chickens [50]. The application of levamisole to improve poultry immunity is indicated in low feed quality and stressful conditions [51]. Experimental infection of sporulated Coccidian oocysts in 170-day-old chickens along with oral administration of levamisole as an immunomodulatory compound demonstrated better weight gain, lesser mortality and morbidity, as well as a higher antibody response in the treatment group compared to the control group. It was revealed that when levamisole was used alone, without anticoccidial drugs, it could not improve chicken immunity or flock performance, but it promoted flock performance when used in combination with anticoccidial drugs [52]. Moreover, Newcastle disease (ND)/avian influenza (AI)-oiled vaccine administration along with levamisole oral usage revealed better immunity in ducklings. Serum IgG titers and the secretion of both Th1- and Th2-related cytokines increased in levamisole-treated groups compared with control, which indicated that levamisole is a useful adjuvant [53]. It has also been shown that levamisole administration might reduce the destructive effects of copper toxicity on the immune system in broiler chickens [54]. Altogether, immunostimulating effects of levamisole were detected in poultry, which might clarify levamisole administration in immunosuppressive diseases such as infectious bursal and ND.

Levamisole’s impact on the human immune system has been the subject of numerous studies over the years. The greatest effect of levamisole on the treatment of human atopic diseases has been suggested with reduced symptoms, including sneezing, itching, nasal congestion, and redness of the eyes, by reduction of cytokine sensitization [55]. The impact of levamisole on innate immunity has shown that levamisole can increase the expression of CD80, CD86, and CD83 as well as the human leukocyte antigen D-correlated receptor on the cell membrane of dendritic cells and, as a result, increase the stimulation of Th1 cells [13]. The influence of levamisole on humoral immunity has been examined by inducing Th2 and T cytotoxic cells [13,56]. Many studies have suggested that the impact of levamisole on atopic diseases can also have an influence on the ratio between Th (1/2) cells as well as cytokine status [55,57]. Moreover, the impact of co-administration of levamisole on the human immunodeficiency virus-1 vaccine in mice indicated an improved vaccine response by reducing cytokine production [58]. Combinations of levamisole with antibacterial drugs have been suggested for the treatment of chronic bacterial diseases, including brucellosis [59,60] and *Escherichia coli* infections [61]. The protective effect of levamisole against bacterial infections has been shown, which is related to the enhancement of monocyte phagocytosis and T3 cell numbers [62]. According to the mentioned studies, levamisole could be used as an immunostimulative drug in combination with other antibacterial as well as anti-viral drugs. However, further study is recommended for detecting the exact mechanism of levamisole in human immunity.

### Levamisole used for adjuvant therapy in vaccination programs

Neonatal humans and animals are very vulnerable to microbial pathogens, and their immunity mostly depends on the effectiveness of passive immunity [63]. The survival rate during this period depends on the neonatal adaptation to extrauterine life. Passive immunity is mainly transferred by colostrum in mammalian species. Colostrum consumption by neonates and IgG transfer to them are indicated as the first immunity defense system for neonates.

Immunoglobulin concentrations in colostrum are suggested to be related to the mortality and morbidity of calves with pneumonia [64]. At the early stage of life, passive immunity effectively reduces disease status by promoting immunoglobulin levels in the first weeks of life [65]. Immaturity of the immune system in neonates is an indication for levamisole use to protect the gastrointestinal tract, especially against various strains of *E. coli*. The combination of adjuvant with various bacterial vaccines improved the protective potential of vaccination, especially in the early stages of life [61]. Levamisole has been used as an oral adjuvant for stimulation of the mucosal immune system [41]. Intensive studies have been conducted recently to evaluate protocols of levamisole administration for nonspecific immunoprophylaxis in neonatal vaccination [41,63]. The protective effect of levamisole was observed in weaned pigs that are infected with the F4ac+ enterotoxigenic *E. coli* strain, as indicated by the observation of intestinal villous M cells and the assessment of IgA levels.

The combination of levamisole and vitamin E has been utilized as an immune system stimulator in calves. Its beneficial effect has been observed in promoting the humoral immunity of calves and enhancing their immunity against neonatal infections in the field [31]. The effects of a combination of trace elements and vitamins with levamisole on the immunity of 135-day-old chicks were examined in coccidial vaccination, which resulted in better weight gain in chickens, higher antibody titers, and lower mortality [52,66]. Furthermore, the protective effect of this combination has been suggested against aflatoxicosis in broiler chickens [67]. Moreover, levamisole was administered to 29-day-old chicks for 3 days, and its protective effect was detected by a delayed-type hypersensitivity reaction test. Levamisole not only increased cellular immune response but also improved flock performance parameters when co-administered with the infectious bursal disease vaccine [43]. Irmak et al. [44] evaluated the effect of concomitant oral administration of levamisole with routine medications on treating chronic brucellosis in humans. The results showed that all patients in the levamisole group showed improvement, and there was a significant difference in the 6-month treatment process between the treatment and control groups. The assessment of blood parameters showed that levamisole increased the immune cell status and immunity response in chronic brucellosis [44]. In addition, co-administration of levamisole with the *Brucella* vaccine has been suggested for the enhancement of humoral and cell-mediated immunity [45]. According to the mentioned studies, levamisole as an adjuvant has had a significant effect on the performance of vaccines in humans and farm animals. Examining the effect of levamisole on viral vaccines is the same as on bacterial vaccines, and the use of levamisole can be recommended as an adjuvant based on recent studies.

### The effect of levamisole administration on endometriosis

Endometriosis, a medical condition represented by the growth of tissues resembling the lining of the womb in locations outside the uterus, has been recognized for numerous decades and is not considered a condition that resolves on its own. However, aberrant active and passive immune responses seem to be essential for ectopic endometrial proliferation. Unfortunately, the endometriosis pathophysiology is not very clear, and it is believed that endometrial cells evade apoptosis and the immunosurveillance system in the peritoneal cavity [65]. Recent studies have revealed that several factors contribute to endometriosis, including reduced T cell reactivity and natural killer cytotoxicity, activation of B cells, elevated antibody production, increased number and activation of peritoneal macrophages, and alterations in inflammatory mediators [46]. Levamisole was used in experimental endometriosis, either alone or in combination with other agents, as a treatment approach. The experimental and randomized human-based studies revealed a reduction in endometriosis following levamisole treatment. Reports suggest the potential efficacy of levamisole in preventing and treating endometriosis, with indications that it may be as efficient as leuprolide in causing retrogression of endometriotic implant volume [68,69]. Further studies are recommended for clarifying the main levamisole mechanism of action in endometriosis. Also, the combination of levamisole with other immunoprotective agents could be investigated for reducing the endometriosis rate in farm animals and human papulations.

### Anticancer potential of levamisole

**Figure 2.**
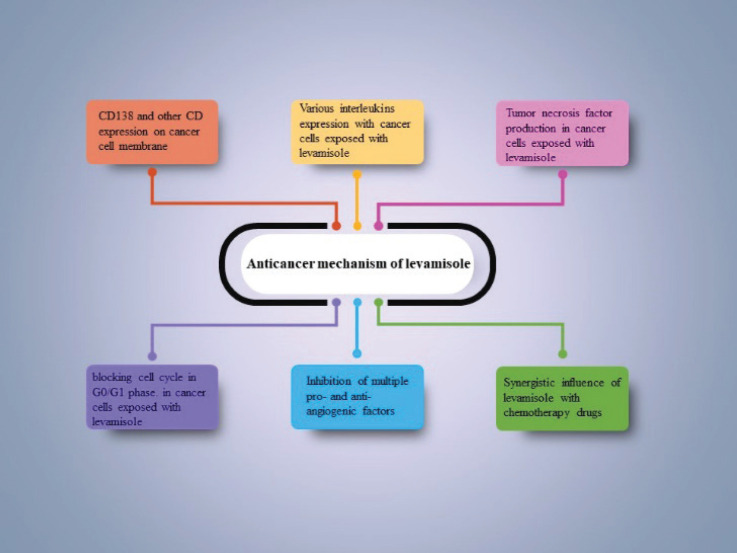
The main anticancer mechanism suggested for levamisole and its derivatives.

Many studies have examined the anti-cancer and adjuvant effects of levamisole in the treatment of various cancers (Fig. 2) [66,^67^,70]. Recent research has investigated the immunomodulatory effects of levamisole in combination with chemotherapy on the expression of specific anticancer markers. It has shown a potential reduction in breast tumor development through the induction of tumor necrosis factor-related apoptosis-inducing ligand (TRAIL) [71]. Levamisole was used in combination with taurine for the prevention of Ehrlich ascites in carcinoma-bearing infected mice. This combination reduced tumor cell development and potentiated the efficacy of anticancer chemotherapy by reducing their immunosuppressive adverse effects [67]. In an *in vitro* evaluation of levamisole on multiple myeloma, the rate of growth of the cell lines decreased in a dose-dependent pattern. Expression of CD138 on the cancer cell membrane and interleukin-6 secretion from the cell for apoptosis inhibition suggested that levamisole could be used as a supplementary treatment for multiple myeloma [72]. Levamisole also induced apoptosis by inducing tumor necrosis TRAIL in five human lung cancer cell lines (HCC827, H1975, H157, H460, and A549), which showed the antitumor potential of levamisole. Moreover, *in vivo* assessment highlighted the blocking effect of levamisole on the cell cycle in the G0/G1 phase of lung cancer cell lines [70]. Furthermore, levamisole has been utilized as an adjuvant to a chemotherapy regimen in colon cancer treatment and has improved the healing process and reduced tumor proliferation [73]. Levamisole has also been utilized as an immunomodulator for canine malignant mammary neoplasms [71]. Co-administration of levamisole with 5-fluorouracil is also suggested for various cancer therapies, such as colon cancer; however, long-term administration of levamisole with a high dosage caused various side effects, including vomiting, which prevented levamisole usage in all patients [74]. The effectiveness of levamisole and Tarantula cubensis, a homeopathic drug derived from spider venom, as anti-tumor treatments for teat papillomatosis in cows was compared. Histopathological and antioxidant evaluations demonstrated the anticancer properties of levamisole. The levamisole treatment group showed significant growth inhibition. However, it was reported that the Tarantula cubensis extract exhibited even higher effectiveness in treating teat papillomatosis compared to levamisole [75].

Angiogenesis is recognized as a complex process involving numerous angiogenic factors. Notably, tumors heavily rely on angiogenesis, and inhibiting this process is considered a potent strategy for cancer treatment [76]. Several studies have been conducted for the assessment of multiple aspects of the anti-angiogenic properties of levamisole [77–79]. Friis et al. [80] showed that levamisole can inhibit the angiogenesis of tumors *in vitro* and *in vivo* by inhibiting vascular endothelial growth factor. Moreover, it was suggested that levamisole mimics the function of nonsteroidal anti-inflammatory drugs and inhibits endothelial cell proliferation, migration, and tube formation [80]. Wang et al. [81] evaluated the effect of levamisole on an ovarian cancer cell line. They observed that levamisole exhibited a substantial effect on decreasing the rate of proliferation and growth of ovarian cancer cells in a dose-dependent manner [81]. Concomitant use of levamisole with common anticancer drugs such as chlorambucil, leucovorin, and fluorouracil has been shown to be strongly synergistic, and as a result, researchers have recommended the concomitant use of levamisole with anticancer agents [82,83]. Collectively, levamisole’s anticancer potential has been detected in various studies in combination with other chemotherapy and anticancer drugs. Based on the mentioned studies, levamisole alone didn’t have a high protective effect as an anticancer drug, and the synergistic effect of levamisole is more important in anticancer treatment. However, the anti-angiogenic capability of levamisole could be considered the main tumor inhibitory factor, as mentioned in *in vitro* and *in vivo* research indicating multiple aspects of the anti-angiogenic properties of levamisole [77–79].

### Antiviral effects of levamisole supplementation

A number of studies have evaluated the efficacy of levamisole as an immunostimulant against viruses [84,85]. They applied levamisole in two main ways: as an adjuvant with vaccines and as a supplementary therapy [84].

Traditionally, vaccines are often formulated with oil used as an adjuvant. Nowadays, new ingredients, especially immunostimulatory compounds, have been used as adjuvants to prevent infectious diseases and improve the benefits of vaccination programs [86]. Many studies have shown that using levamisole with the hepatitis vaccine can increase the immunogenicity of the vaccine and decrease its adverse effects [87,88]. Furthermore, in poultry production, the effects of levamisole as an adjuvant on the efficacy of infectious bursal disease, Newcastle, and AI (H5N1) vaccines have been studied [43,^89^,90].

Interestingly, a number of studies have indicated the beneficial effect of levamisole on foot and mouth disease (FMD) in animals [91,92]. The immunostimulatory effect of levamisole was demonstrated in buffaloes vaccinated with FMD serotypes O, A, and SAT2 [91]. Co-administration of levamisole with the FMD vaccine enhanced humoral and cell-mediated immunity in vaccinated sheep at 8 and 14 weeks after levamisole use [92]. In another study, levamisole significantly increased antibody titers in serum; however, the colostral antibody was not significantly different in the control and experiment groups [93].

Recently, the effects of levamisole on the treatment, control, and management of COVID-19 have been examined. COVID-19, a global disease with high morbidity and mortality, occurs with severe acute respiratory symptoms. It affects multiple organs, particularly the lungs, and manifests with pulmonary symptoms, including acute lung injury and acute respiratory distress syndrome. In addition, COVID-19 infects other systems, including the cardiac and nervous systems, as well as causing pancreatic malfunction and kidney failure [94,95]. The systemic manifestation of COVID-19 is linked to the extensive dispersion of angiotensin-converting enzyme 2 receptors throughout the body [96]. Most studies indicated that the main effect of levamisole was on the respiratory system, as seen by its *in vitro* use on lung cancer cell lines [70]. Moreover, inhibition of papain-like protease activity in the coronavirus cell cycle as well as activation of glucocorticoid receptors for the protection of the kidney are suggested as the mechanisms by which levamisole protects against COVID-19 [97]. Importantly, the beneficial effects of levamisole were reported for the reduction of viral diarrhea in patients infected with COVID-19 [98]. Levamisole has been orally administered to outpatients with COVID-19. Levamisole increased the general health condition and decreased the rate of developing fever, chills, and myalgia. However, during therapy, there was a nonsignificant alteration in dyspnea, cough, diarrhea, nausea, vomiting, sore throat, hyposmia, dysgeusia, and anorexia compared to the control group [99]. Interestingly, levamisole derivatives could be used as the main drug for COVID-19 treatment because they contain the molecule N°6 structure in levamisole, which could be further developed to stop the COVID-19 pandemic [100]. Altogether, it can be concluded that levamisole and its derivatives can be used in the future as antiviral drugs. Utilizing levamisole has been suggested as a complementary therapy in the management of viral diseases, but levamisole should be taken cautiously to avoid its side effects in susceptible patients. Some studies have reported skin reactions in patients who were allergic to levamisole. Skin reactions also occurred in cases of levamisole poisoning [101].

## Conclusion

It seems that levamisole is widely used as an anthelminthic, anti-cancer, anti-viral, and immune system-inducing agent in humans and animal species. In the articles examined, various mechanisms have been proposed for levamisole immunoprotective effects, but hormonal alteration and stress hormone reduction were indicated as the main mechanisms in various animal species. More investigation is required to explore the variety of levamisole functions and its potential applications in the prevention and treatment of diseases in humans and a wide range of animal species.

## List of abbreviations

COVID -19, coronavirus disease of 2019; WBC, white blood cell; Ig, immunoglobulin; ND, Newcastle disease; AI, avian influenza; Th1, T helper 1; IL, interleukin; TRAIL, TNF-related apoptosis-inducing ligand; FMD, foot and mouth disease.

## References

[1] Brooker S. (2010). Estimating the global distribution and disease burden of intestinal nematode infections: adding up the numbers—a review. Int J Parasitol.

[2] Macpherson CN. (2005). Human behaviour and the epidemiology of parasitic zoonoses. Int J Parasitol.

[3] Mabbott NA. (2018). The influence of parasite infections on host immunity to co-infection with other pathogens. Front Immunol.

[4] Harizanov R, Rainova I, Tsvetkova N, Kaftandjiev I, Borisova R, Ivanova A (2020). Prevalence of intestinal parasitic infections among the Bulgarian population over a three year period (2015-2017). Helminthologia.

[5] Brito LL, Barreto ML, Silva Rde C, Assis AM, Reis MG, Parraga I (2003). Risk factors for iron-deficiency anemia in children and adolescents with intestinal helminthic infections. Rev Panam Salud Publica.

[6] Doran S, Abuamr KM. (2018). Intestinal parasite as cause of abdominal pain and diarrhea in a patient with normal labs and stool studies. Am J Gastroenterol.

[7] Zavala GA, Doak CM, Portrait F, Seidell JC, Garcia OP, Rosado JL (2019). Are intestinal parasites associated with obesity in Mexican children and adolescents?. Parasitol Int.

[8] Martin RJ, Robertson AP, Buxton SK, Beech RN, Charvet CL, Neveu C. (2012). Levamisole receptors: a second awakening. Trends Parasitol.

[9] World Health Organization (2019). World Health Organization model list of essential medicines: 21st list 2019.

[10] Mauger M, Kelly G, Annandale C, Robertson I, Waichigo F, Aleri J. (2022). Anthelmintic resistance of gastrointestinal nematodes in dairy calves within a pasture-based production system of south West Western Australia. Austr Vet J.

[11] Weng CH, Liu ZC. (2019). Drug-induced anti-neutrophil cytoplasmic antibody-associated vasculitis. Chin Med J (Engl).

[12] Soudkolaei AS, Kalidari GA, Borji H. (2021). Anthelmintic efficacy of fenbendazole and levamisole in native fowl in northern Iran. Parasit Vectors.

[13] Chen LY, Lin YL, Chiang BL. (2008). Levamisole enhances immune response by affecting the activation and maturation of human monocyte-derived dendritic cells. Clin Exp Immunol.

[14] Wiebke EA, Grieshop NA, Loehrer PJ, Eckert GJ, Sidner RA. (2003). Antitumor effects of 5-fluorouracil on human colon cancer cell lines: antagonism by levamisole. J Surg Res.

[15] Ejlertsen B, Mouridsen HT, Jensen MB, Andersen J, Andersson M, Kamby C (2010). Cyclophosphamide, methotrexate, and fluorouracil; oral cyclophosphamide; levamisole; or no adjuvant therapy for patients with high-risk, premenopausal breast cancer. Cancer.

[16] Roostaei Firozabad A, Meybodi ZA, Mousavinasab SR, Sahebnasagh A, Jelodar MG, Karimzadeh I (2021). Efficacy and safety of levamisole treatment in clinical presentations of non-hospitalized patients with COVID-19: a double-blind, randomized, controlled trial. BMC Infect Dis.

[17] Blecha F. (2001). Immunomodulators for prevention and treatment of infectious diseases in food-producing animals. Vet Clin North Am Food Anim Pract.

[18] Naylor PH, Hadden JW. (2003). T cell targeted immune enhancement yields effective T cell adjuvants. Int Immunopharmacol.

[19] Biller-Takahashi J, Montassier H, Takahashi L, Urbinati E. (2016). Levamisole promotes an adjuvant effect on the immunity of pacu (*Piaractus mesopotamicus*) when immunized with *Aeromonas hydrophila*, even when provided in the diet. Anim Feed Sci Technol.

[20] Kumari J, Sahoo PK. (2006). Dietary levamisole modulates the immune response and disease resistance of Asian catfish *Clarias batrachus* (Linnaeus). Aquac Res.

[21] Singh CS, Parine NR, Swain SM, Pandey S, Bobbarala V. (2010). Immunomodulatory effects of dietary intake of levamisole on the immune system of common carp (*Cyprinus carpio*) and control of *Aeromonas hydrophila* infection in ponds. Pharm Res.

[22] Yuji Sado R, de Almeida Bicudo AJ, Possebon Cyrino JE. (2010). Dietary levamisole influenced hematological parameters of juvenile pacu, *Piaractus mesopotamicus* (Holmberg 1887). J World Aquac Soc.

[23] Ispir U, Yonar M. (2007). Effects of levamisole on phagocytic activity of rainbow trout (*Oncorhynchus mykiss* W.). Acta Vet Brno.

[24] Cuesta A, Esteban M, Meseguer J. (2002). Levamisole is a potent enhancer of gilthead seabream natural cytotoxic activity. Vet Immunol Immunopathol.

[25] Gopalakannan A, Arul V. (2006). Immunomodulatory effects of dietary intake of chitin, chitosan and levamisole on the immune system of *Cyprinus carpio* and control of *Aeromonas hydrophila* infection in ponds. Aquaculture.

[26] Hang BTB, Phuong NT, Kestemont P. (2014). Can immunostimulants efficiently replace antibiotic in striped catfish (*Pangasianodon hypophthalmus*) against bacterial infection by *Edwardsiella ictaluri*?. Fish Shellfish Immunol.

[27] Li G, Guo Y, Zhao D, Qian P, Sun J, Xiao C (2006). Effects of levamisole on the immune response and disease resistance of *Clarias fuscus*. Aquaculture.

[28] Sadati NY, Youssefi MR, Hosseinifard SM, Tabari MA, Giorgi M. (2021). Pharmacokinetics and pharmacodynamics of single and multiple-dose levamisole in belugas (*Huso huso*): main focus on immunity responses. Fish Shellfish Immunol.

[29] Pahor-Filho E, Castillo ASC, Pereira NL, Pilarski F, Urbinati EC. (2017). Levamisole enhances the innate immune response and prevents increased cortisol levels in stressed pacu (*Piaractus mesopotamicus*). Fish Shellfish Immunol.

[30] Mahendra K, Jana S. (2019). Immunomodulatory effect of the consciousness energy healing treated novel test formulation. Alt Med Chiropractic OA J.

[31] Pekmezci D, Cakiroglu D. (2009). Investigation of immunmodulatory effects of levamisole and vitamin E on immunity and some blood parameters in newborn Jersey calves. Vet Res Commun.

[32] Mohri M, Seifi H, Zamani Sani S. (2005). Effects of oral administration of levamisole on non-specific immunity, serum proteins and health in normal colostrum-fed neonatal dairy calves. Comp Clin Path.

[33] Mushtaq M, Agrawal R, Singh R, Pande N. (2019). Immunomodulatory effect of levamisole therapy in pre-parturient dairy cows. Intas Polivet.

[34] Duarte KMR, de Melo A, Gomes L, Farias J, Poncio V, Alvarez R. (2019). Levamisole-phosphate used in dairy cattle on pre-parturient and reproductive performance during post-parturient period. J Agric Vet Sci.

[35] Pancarci SM, Gurbulak K, Oral H, Karapehlivan M, Tunca R, Colak A. (2009). Effect of immunomodulatory treatment with levamisole on uterine inflammation and involution, serum sialic acid levels and ovarian function in cows. Kafkas Univ Vet Fak Derg.

[36] Darwish AA, Eldakroury MF. (2023). Clinicopathological evaluation of some immunostimulants‘ effects in Barki lambs. Iraqi J Vet Sci.

[37] Dabbir BKR, Nanjundaiah K. (2020). Enhancement of immunity of sheep pox vaccinations with levamisole and bioplex. J Bio Innov.

[38] Rashid BM, Yüksek N. (2019). The effects of immunostimulants (zinc, levamisole, vitamin AD3E) use together with enterotoxemia vaccine on immunoglobulins in sheeP. Turk J Vet Res.

[39] Božić F, Bilić V, Valpotić I. (2003). Levamisole mucosal adjuvant activity for a live attenuated *Escherichia coli* oral vaccine in weaned pigs. J Vet Pharmacol Ther.

[40] Chethan GE, Kumar DU, Garkhal J, Sircar S, Malik YPS, Sahoo NR (2019). Immunomodulating dose of levamisole stimulates innate immune response and prevents intestinal damage in porcine rotavirus diarrhea: a restricted-randomized, single-blinded, and placebo-controlled clinical trial. Trop Anim Health Prod.

[41] Janjatović A, Lacković G, Božić F, Popović M, Valpotić I. (2008). Levamisole synergizes proliferation of intestinal IgA+ cells in weaned pigs immunized with vaccine candidate F4ac+ nonenterotoxigenic *Escherichia coli* strain. J Vet Pharmacol Ther.

[42] Valpotić H, Šperanda M, Kovšca-Janjatović A, Ðidara M, Lacković G, Božić F (2014). Levamisole stimulates proliferation of circulating and intestinal immune cell subsets, gut health and performance in weaned pigs. Can J Anim Sci.

[43] Bilandžić N, Šimić B, Terzić S, Žurić M. (2010). The influence of levamisole on cortisol concentration and peripheral blood in artificially stressed pigs. Vet Arhiv.

[44] Dair HF, Ali AM. (2016). Immunomodulatory effects of levamisole hydrochloride and *Nigella sativa* against infectious bursal disease (IBD) in chicks. Microbiol Res J Int.

[45] Irmak H, Buzgan T, Karahocagil MK, Evirgen O, Akdeniz H, Demiroz AP. (2003). The effect of levamisole combined with the classical treatment in chronic brucellosis. Tohoku J Exp Med.

[46] Mansour AM. (2018). Effect of levamisole administration on immunogenic and protective capacity of *Brucella abortus* RB51. Natl J Physiol Pharm Pharmacol.

[47] Riccio L, Santulli P, Marcellin L, Abrao MS, Batteux F, Chapron C. (2018). Immunology of endometriosis. Best Pract Res Clin Obstet Gynaecol.

[48] Sajid M, Iqbal Z, Muhammad G, Iqbal M. (2006). Immunomodulatory effect of various anti-parasitics: a review. Parasitology.

[49] Riehemann K, Schneider SW, Luger TA, Godin B, Ferrari M, Fuchs H. (2009). Nanomedicine—challenge and perspectives. Angew Chem Int Ed Engl.

[50] Klasing KC. (2007). Nutrition and the immune system. Br Poult Sci.

[51] Saeed M, Naveed M, Leskovec J, Kakar I, Ullah K, Ahmad F (2020). Using Guduchi (*Tinospora cordifolia*) as an eco-friendly feed supplement in human and poultry nutrition. Poult. Sci.

[52] Ahmad T, Hussain M, Yousaf A, Masood M, Muhammad G. (2018). Effects of vitamin E, selenium and levamisole on immune response of broiler birds artificially infected with coccidiosis. Vet World.

[53] Zhang Y, Chen H, Zeng X, Wang P, Li J, Wu W. (2014). Levamisole enhances immunity in ducklings vaccinated against *Riemerella anatipestifer*. Microbiol Immunol.

[54] Yigit AA, Cinar M, Yildirim E. (2012). The effects of levamisole on oxidative stress induced by copper intoxication in broilers. N Z Vet J.

[55] Kocabas CN, Sekerel BE, Firat PA, Okur H, Adahoglu G. (2004). Levamisole: might it be used in treatment and prevention of atopic diseases?. J Asthma.

[56] Witonsky S, Buechner-Maxwell V, Santonastasto A, Pleasant R, were S, Wagner B (2019). Can levamisole upregulate the equine cell-mediated macrophage (M1) dendritic cell (DC1) T-helper 1 (CD4 Th1) T-cytotoxic (CD8) immune response *in vitro*?. J Vet Intern Med.

[57] Szeto C, Gillespie KM, Mathieson PW. (2000). Levamisole induces interleukin-18 and shifts type 1/type 2 cytokine balance. Immunology.

[58] Rastgou R, Rouhollah F. (2020). The role of levamisole and HIV-1 Nef-p24 fusion protein in IL-4 gene expression for evaluating humoral immune response. Arch Adv Biosci.

[59] Dizer U, Hayat L, Beker CM, Gorenek L, Ozguven V, Pahsa A. (2005). The effect of the doxycycline-rifampicin and levamisole combination on lymphocyte subgroups and functions of phagocytic cells in patients with chronic brucellosis. Chemotherapy.

[60] Moudgil AD, Mittra S, Sen D, Agnihotri R, Sharma D. (2015). Biochemical and leucocytic response study of herbal immunomodulators against levamisole in *Toxocara canis* infected mice. Indian J Anim Res.

[61] Božić F, Banović F, Šuran J, Crnić AP. (2011). Adjuvant activity of levamisole for experimental F18ac+ *Escherichia coli* oral vaccine against porcine post-weaning colibacillosis. Vet Arhiv.

[62] Singh N, Singh B, Kumar R. (2023). Effect of uterine lavage, levamisole, PGF2α and its combinations on haematological indices and bacterial load in estrual mucus of endometritic buffaloes. Indian J Vet Sci Biotechnol.

[63] Valpotić H, Janjatović AK, Lacković G, Božić F, Dobranić V, Svoboda D (2010). Increased number of intestinal villous M cells in levamisole-pretreated weaned pigs experimentally infected with F4ac+ enterotoxigenic *Escherichia coli* strain. Eur J Histochem.

[64] Singh D, Kumar M, Choudhary P, Singh H. (2009). Neonatal calf mortality-an overview. Intas Polivet.

[65] Berkkanoglu M, Arici A. (2003). Immunology and endometriosis. Am J Reprod Immunol.

[66] Laudisi F, Maronek M, Di Grazia A, Monteleone G, Stolfi C. (2020). Repositioning of anthelmintic drugs for the treatment of cancers of the digestive system. Int J Mol Sci.

[67] Ibrahim HM, Abdel Ghaffar FR, El-Elaimy IA, Gouida MS, Abd El Latif HM. (2018). Antitumor and immune-modulatory efficacy of dual-treatment based on levamisole and/or taurine in Ehrlich ascites carcinoma-bearing mice. Biomed Pharmacother.

[68] Azimirad A, Alborzi S, Kumar PV, Zarei A, Azimirad M. (2013). The effects of levamisole on experimental endometriosis: a randomized controlled trial in a rat model. Arch Gynecol Obstet.

[69] Ocal G, Kokcu A, Cetinkaya MB, Tosun M, Kefeli M, Kandemir B. (2007). Efficacy of levamisole on experimental endometriosis. Int J Gynaecol Obstet.

[70] Qiao X, Wang C, Wang W, Shang Y, Li Y, Ni J (2020). Levamisole enhances DR4-independent apoptosis induced by TRAIL through inhibiting the activation of JNK in lung cancer. Life Sci.

[71] Manikkan Dileepkumar K, Kumar Maiti S, Kumar N, Shams-uz-Zama MM. (2015). Therapeutic evaluation of anti-angiogenic and chemotherapy with or without Cox-2 inhibitor and immunomodulator drug in the management of canine mammary neoplasm. Pak Vet J.

[72] Nageshwari B, Merugu R. (2017). Effect of levamisole on expression of CD138 and interleukin-6 in human multiple myeloma cell lines. Indian J Cancer.

[73] Stenstedt K, Hallstrom M, Johansson I, Ingelman-Sundberg M, Ragnhammar P, Edler D. (2012). The expression of CYP2W1: a prognostic marker in colon cancer. Anticancer Res.

[74] O‘Connell MJ, Sargent DJ, Windschit HE, Shepherd L, Mahoney MR, Krook JE (2006). Randomized clinical trial of high-dose levamisole combined with 5-fluorouracil and leucovorin as surgical adjuvant therapy for high-risk colon cancer. Clin Colorectal Cancer.

[75] Paksoy Z, Gülesci N, Kandemir FM, Dinçel GC. (2015). Effectiveness of levamisole and tarantula cubensis extract in the treatment of teat papillomatosis of cows. Indian J Anim Res.

[76] Abdollahi A, Folkman J. (2010). Evading tumor evasion: current concepts and perspectives of anti-angiogenic cancer therapy. Drug Resist Update.

[77] Chandy ML, Soman C, Kumar SP, Kurup S, Jose R. (2016). Understanding molecular mechanisms in multivariant actions of levamisole as an anti-helminthic, anti-inflammatory, antioxidant, anti-neoplastic and immunomodulatory drug. J Oral Maxillofac Surg Med Pathol.

[78] Friis T, Engel AM, Klein BM, Rygaard J, Houen G. (2005). Levamisole inhibits angiogenesis *in vitro* and tumor growth *in vivo*. Angiogenesis.

[79] Sabell E. (2015). Angiogenic effect of chemicals. Master’s theses.

[80] Friis T, Engel AM, Bendiksen CD, Larsen LS, Houen G. (2013). Influence of levamisole and other angiogenesis inhibitors on angiogenesis and endothelial cell morphology *in vitro*. Cancers (Basel).

[81] Wang YC, Bai MY, Yeh YT, Tang SL, Yu MH. (2020). CD133 Targeted PVP/PMMA microparticle incorporating levamisole for the treatment of ovarian cancer. Polymers (Basel).

[82] Hourdequin KC, Schpero WL, McKenna DR, Piazik BL, Larson RJ. (2013). Toxic effect of chemotherapy dosing using actual body weight in obese versus normal-weight patients: a systematic review and meta-analysis. Ann Oncol.

[83] Salem FS, Badr MO, Neamat-Allah AN. (2011). Biochemical and pathological studies on the effects of levamisole and chlorambucil on Ehrlich ascites carcinoma-bearing mice. Vet Ital.

[84] Kang Y, Jin H, Zheng G, Xie Q, Yin J, Yu Y (2005). The adjuvant effect of levamisole on killed viral vaccines. Vaccine.

[85] Zhang L, Liu Y. (2020). Potential interventions for novel coronavirus in China: a systematic review. J Med Virol.

[86] O‘Hagan DT, MacKichan ML, Singh M. (2001). Recent developments in adjuvants for vaccines against infectious diseases. Biomol Eng.

[87] Alavian SM, Tabatabaei SV. (2010). Effects of oral levamisole as an adjuvant to hepatitis B vaccine in adults with end-stage renal disease: a meta-analysis of controlled clinical trials. Clin Ther.

[88] Fabrizi F, Dixit V, Messa P, Martin P. (2010). Meta-analysis: levamisole improves the immune response to hepatitis B vaccine in dialysis patients. Aliment Pharmacol Ther.

[89] Habibi M, Ghahri H, Zadeh RS, Yeganeh F. (2012). Effects of levamisole on the immune response of broilers against Newcastle disease vaccines. Afr J Pharm Pharmacol.

[90] Sabry E, Arafa A, Ali M, Lamya A. (2010). Immunomodulator effect of levamisole on broilers vaccinated with avian influenza vaccine (H5N1). In 3rd Animal Wealth Research Conference in the Middle East & North Africa, Egypt.

[91] El-Shahedy M, Abdel Fatah S. (2015). Immunostimulant activity of levamisole to polyvalent FMD vaccine in buffaloes. Suez Canal Vet Med J.

[92] Shawky M, Mohamed A, Hind MD, Ekbal MF. (2012). Immunological effect of levamisole as immunostimulant in vaccination with bivalent oil adjuvant foot and mouth disease vaccine in sheep. Zag Vet J.

[93] Qureshi ZI, Lodhi LA, Jamil H, Nawaz M. (2000). Effect of levamisole hydrochloride on serum and colostral antibody titres against foot and mouth disease virus in vaccinated buffaloes (*Bubalus bubalis*). Vet Arhiv.

[94] Al-Kuraishy HM, Al-Gareeb AI, Qusty N, Cruz-Martins N, El-Saber Batiha G. (2021). Sequential doxycycline and colchicine combination therapy in COVID-19: the salutary effects. Pulm Pharmacol Ther.

[95] Sanadgol H, Khoshnoodi M, Mashhadi MA, Forghani MS. (2011). Effect of adding levamisole on seroconversion response to hepatitis B virus vaccination in hemodialysis patients: a single-center experience. Iran J Kidney Dis.

[96] Al-Kuraishy HM, Hussien NR, Al-Naimi MS, Al-Buhadily AK, Al-Gareeb AI, Lungnier C. (2020). Renin–Angiotensin system and fibrinolytic pathway in COVID-19: one-way skepticism. Biomed Biotechnol Res.

[97] Al-Kuraishy HM, Al-Gareeb AI, Alkazmi L, Alexiou A, Batiha GE. (2021). Levamisole therapy in COVID-19. Viral Immunol.

[98] Uyaroğlu OA, Güven GS, Güllü İ. (2020). Can Levamisole be used in the treatment of COVID-19 patients presenting with diarrhea?. J Infect Dev Ctries.

[99] Asgardoon MH, Kazemi-Galougahi MH, Dehnavi AZ, Khodaei B, Behkar A, Dehpour AR (2021). Efficacy of levamisole with standard care treatment vs standard care in clinical presentations of non-hospitalized patients with COVID-19: a randomized clinical trial. BMC Infect Dis.

[100] El Khatabi K, Aanouz I, Alaqarbeh M, Ajana MA, Lakhlifi T, Bouachrine M (2022). Molecular docking, molecular dynamics simulation, and ADMET analysis of levamisole derivatives against the SARS-CoV-2 main protease (M(Pro)). Bioimpacts.

[101] Keim CK, Schwartz RA, Kapila R (2023). Levamisole-induced and COVID-19-induced retiform purpura: two overlapping, emerging clinical syndromes. Arch Dermatol Res.

